# Negative Magnetoresistance in Nanotwinned NiMnGa Epitaxial Films

**DOI:** 10.1038/s41598-018-34057-8

**Published:** 2018-10-24

**Authors:** V. O. Golub, V. A. Chernenko, A. Apolinario, I. R. Aseguinolaza, J. P. Araujo, O. Salyuk, J. M. Barandiaran, G. N. Kakazei

**Affiliations:** 1 Institute of Magnetism NASU and MESU, Kyiv, 03142 Ukraine; 2 BCMaterials & University of Basque Country, P.O. Box 644, Bilbao, 48080 Spain; 3 Ikerbasque, Basque Foundation for Science, Bilbao, 48013 Spain; 4 IFIMUP-IN/Departamento de Fisica e Astronomia, Universidade do Porto, 4169-007 Porto, Portugal

## Abstract

Magnetic shape memory alloys are under intensive investigation due to their unusual physical properties, such as magnetic shape memory effect, magnetic field induced superelasticity, direct and inverse magnetocaloric effect etc., promising for novel applications. One of the intriguing properties of these materials in a single phase state is a giant magnetoresistance. Here we report the remarkable results about the magnetoresistive properties of epitaxial films of Ni_52.3_Mn_26.8_Ga_20.9_ magnetic shape memory alloy in the temperature range of 100–370 K, well below the martensitic transformation temperature. It was found that the formation of non-collinear magnetic structure due to a nanotwinning of the film results in electron scattering on such a structure and noticeable negative magnetoresistance in the entire investigated temperature range.

## Introduction

The Heusler type magnetic shape memory alloys have been intensively studied during the last decades both due to their quite unusual physical properties and potential applications in different areas of engineering and technology^1^. The interest to these materials was stimulated by the discovery of the magnetic shape memory (MSM) effect consisting in a dramatic size change (up to 12%) under a magnetic field, as a result of twin boundary motion in the martensitic state^2,3^. Since that a number of interesting physical effects have been revealed in these materials, such as the magnetic field induced superelasticity, direct and inverse magnetocaloric effect, exchange bias etc. (see, e.g.^4–7^, and references therein). Note, that according to the theoretical predictions, some magnetic shape memory alloys can exhibit up to a hundred percent of spin polarization, which is very attractive for spintronic applications^7^.

One of the intriguing properties of these materials is negative magnetoresistance (MR) observed near the martensitic transformation. In the heterogeneous magnetic/non-magnetic films, either multilayered^8^ or granular^9^, the MR consists in the resistivity change due to the reduction of magnetic disorder caused by a magnetic field. For a homogeneous ferromagnetic material with localized spins, one can expect MR temperature dependence exhibiting a peak at Curie temperature and going to zero at low temperatures^10^. Such behavior is commonly observed for many Heusler alloys, whereas the overall temperature dependence of magnetoresistance is much more complex (see, e.g.^11,12^, and references therein) and does not exhibit a regular dependence on the phase state of the alloys^11^. A number of reports were focused on investigation of the magnetoresistance at the martensitic transformation, whereby the largest negative magnetoresistance value 60–70% was reported for NiMnIn alloys^13,14^.

There are different (sometimes controversial) explanations in the literature concerning the magnetoresistance in the martensitic and austenitic phases, as well as at the martensitic transformation. The following three main mechanisms were used to explain the experimental magnetoresistance data obtained in Heusler MSM alloys: anisotropic magnetoresistance, intrinsic magnetic scattering and interface spin scattering^11,15,16^. The influence of magnetic field on the crystalline, electronic and magnetic structure in these materials was discussed in refs^11–17^. Particularly, in MSM single crystals, either in a single martensitic or in a single austenitic state, the negative magnetoresistance is considered to be related to the *s*-electron scattering by the localized *d*-spins disordered either due to thermal fluctuations or due to some weak antiferromagnetic exchange in such systems^10,12^. In most cases, electron scattering from the domain walls can be neglected due to their low concentration. For some MSM alloys in martensitic state, the twin boundaries rearrangement under the action of magnetic field is considered as a mechanism of the resistivity change (see e.g.^11^), but it has been argued^18^ that the resistivity of a single crystal is not related to the concentration of twin boundaries. Nevertheless, it will be shown in the present work that the existence of stable fine twin structure can lead to the negative magnetoresistance in the Heusler MSM alloys in the entire temperature range of the martensitic state stability. This effect can be observed for a lot of different MSM alloys, where the value of magnetoresistance can be much higher, in a wide temperature range.

## Results

**Figure 1:**
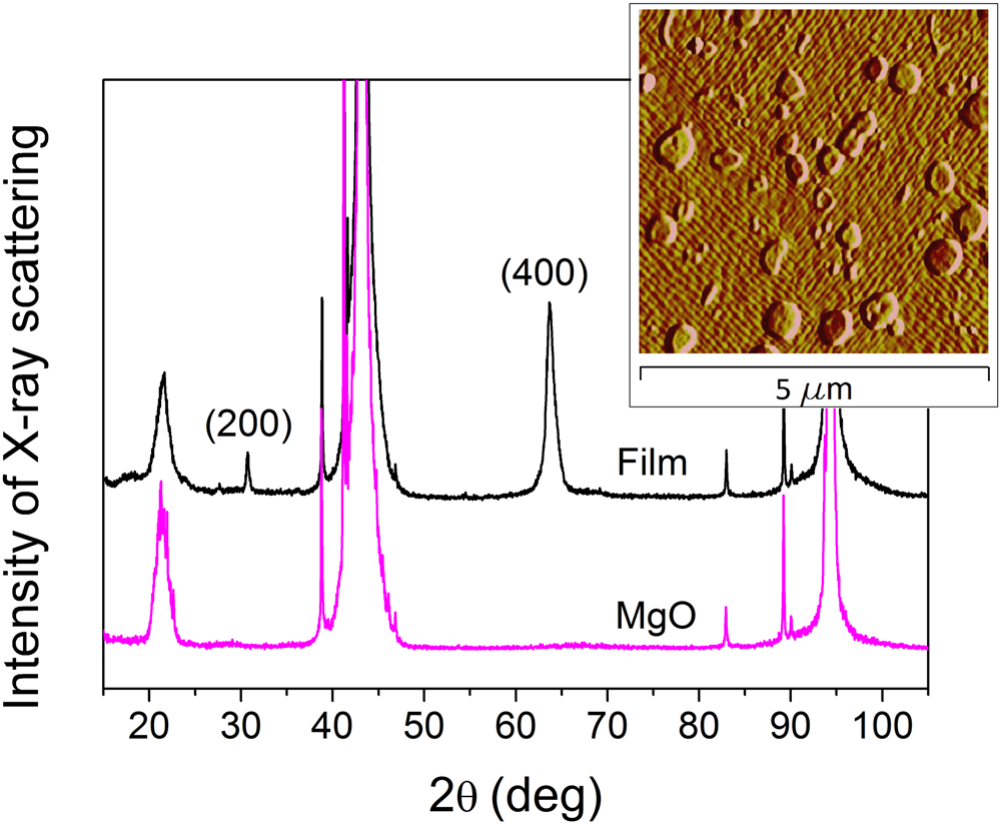
XRD pattern for Ni-Mn-Ga/MgO(001) thin film at room temperature showing (020) and (040) peaks of the orthorhombic unit cell for the film and the reflections belonging to the substrate. Atomic force microscopy image of the film surface is shown in the inset.

A 300 nm-thick Ni_52.3_Mn_26.8_Ga_20.9_ (at.%) film was epitaxially grown by magnetron sputtering onto a MgO(001) substrate. The composition of the film and sample preparation procedure was the same as for the samples investigated in^19^. The composition and the preparation procedure were chosen to obtain epitaxial films with the martensitic transformation temperature well above the Curie temperature to get rid of any influence of the transformation effects on magnetoresistance. Composition of the film was determined with an uncertainty of 0.5 at.% by energy-dispersive x-ray spectroscopy. The cubic-to-orthorhombic martensitic transformation was observed by x-ray diffraction at about 420 K. The x-ray analysis revealed orthorhombic crystal structure with the longest, *a*, and shortest, *c*, axes lying in the film plane, while *b* is perpendicular to the film plane (Fig. 1). The atomic force microscopy image demonstrates a formation of fine stripe-like twin structure with a characteristic twin width, *l*, of about sixty nanometers (see the Inset to Fig. 1). This morphology is similar to the one observed in^19^. Magnetic susceptibility and ferromagnetic resonance (FMR) measurements showed that the film exhibits a ferromagnetic ordering with the Curie temperature around 370 K.

**Figure 2:**
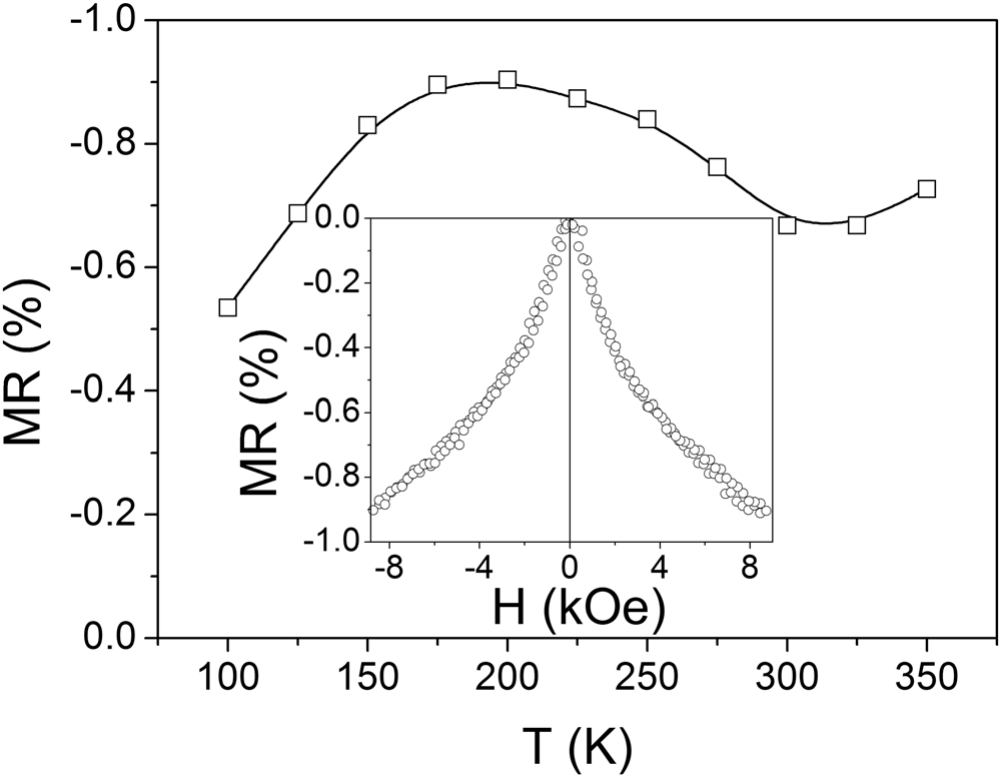
Temperature dependence of the magnetoresistance, *MR*, under magnetic field, *H* = 9 kOe. The dependence of *MR* on magnetic field at *T* = 200 K is shown in the Inset.

**Figure 3:**
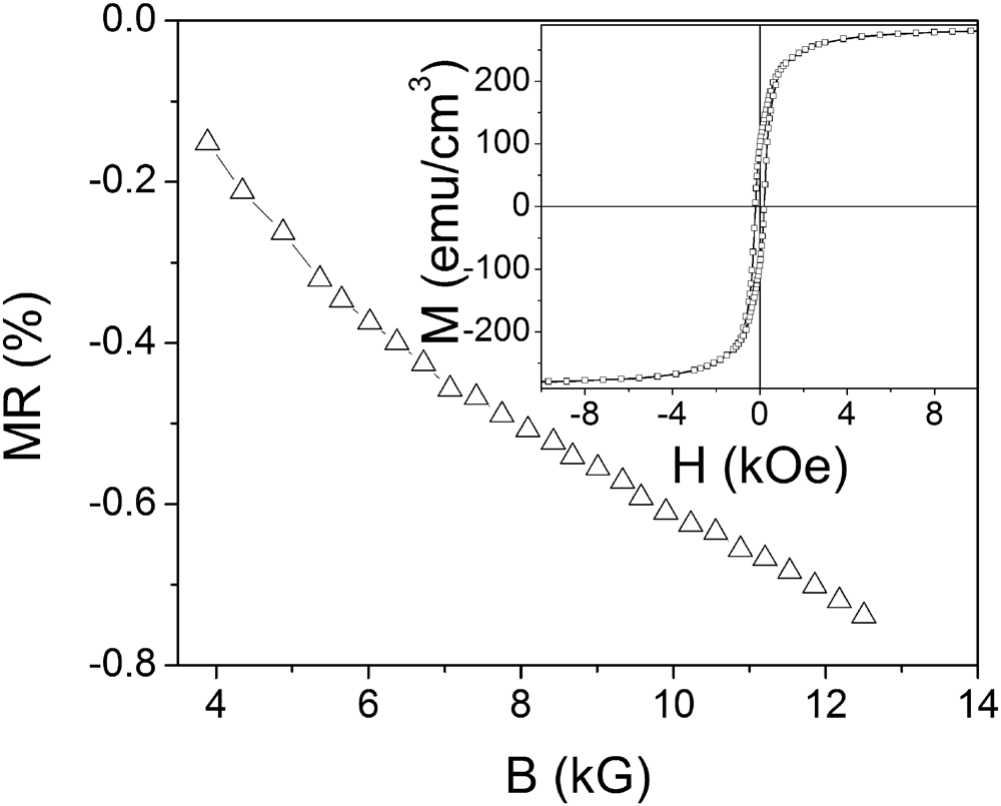
Dependence of *MR* on magnetic field and hysteresis loop of the film (in the Inset) at *T* = 300 K.

The magnetoresistance was measured using a standard four-probe method under magnetic fields up to 9 kOe in the temperature range of 100–370 K. The magnetic field was applied in the film plane. The data are presented in Fig. 2. A negative magnetoresistance with similar dependences on the applied magnetic field, as depicted in the Inset to Fig. 2, is observed in the entire temperature range. The comparison with the magnetization loop shown in the Inset to Fig. 3 reveals that for high field values (above the hysteresis area, *H* > 1 kOe) the variation of the magnetoresistance with the magnetic field follows the change of magnetic induction *B* = *H* + 4π*M* (Fig. 3), which is typical for *s-d* scattering mechanism^10^, where *M* is the magnetization. Noteworthy, the temperature behavior of *MR* in Fig. 2 is non-monotonic. Two broad peaks are observed: the first one is in the vicinity of the Curie temperature and the second one is around 200 K. The first peak can be ascribed to the conduction *s*-electrons scattering by localized *d*-spins which are disordered due to thermal fluctuations, whereas the appearance of the second one will be discussed below.

## Discussion

The origin of the appreciable values of magnetoresistance in wide temperature range can be deduced from the following lines of reasoning. As it has been mentioned above, electron scattering from domain walls can be excluded from the consideration due to their low concentration in this film. The additional confirmation of this statement is that the character of resistance behaviour practically does not change for low fields (below 1 kOe, where the hysteresis behaviour of the magnetization is observed) and for high magnetic fields (up to 9 kOe). The structural characterization does not reveal any additional phase in the films. Only a single martensitic phase is observed below the Curie temperature, which is, in turn, encountered below the martensitic transformation temperature. Magnetic measurements showed no signs of the presence of antiferromagnetic clusters in the film. The presence of such clusters usually results in the shift of hysteresis loop at low temperatures due to the exchange bias effect (see, e.g.^20^). These facts allow excluding the mechanism of scattering from the phase or cluster interfaces. Temperature behaviour of magnetoresistance can not be also ascribed to the spin fluctuation resulting from a weak antiferromagnetic exchange in the system, due to a small manganese ions excess in the alloy^12^.

**Figure 4:**
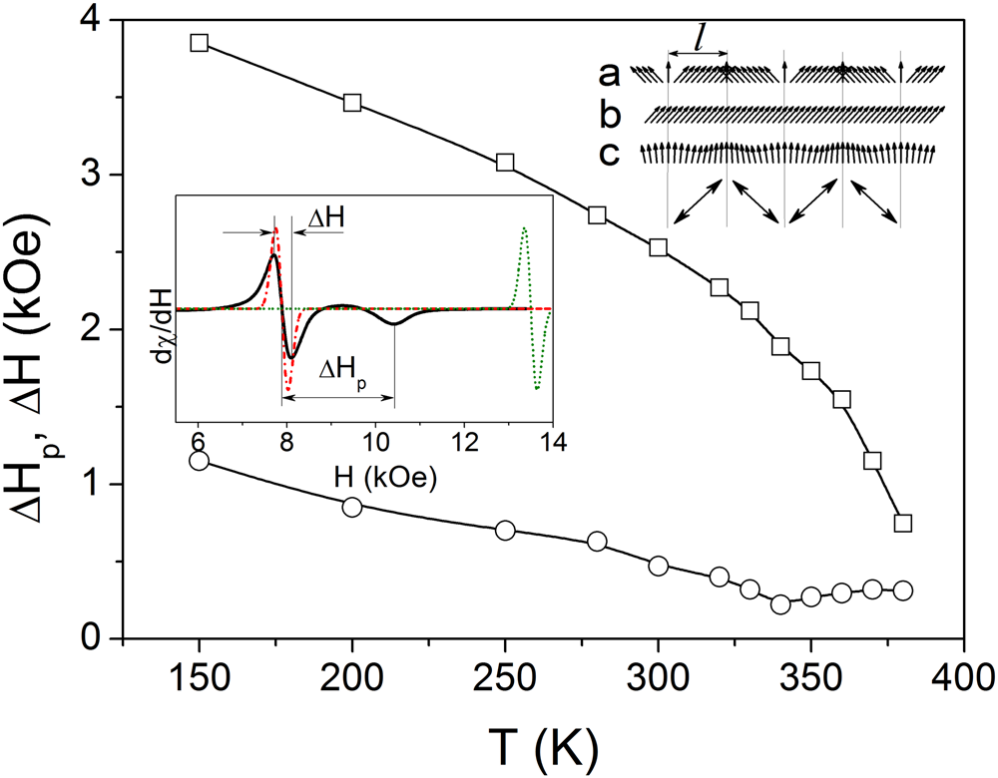
Dependencies of the ferromagnetic resonance line parameters Δ*H*_p_ (□) and Δ*H* (○) on the temperature. Right-up corner area shows a schematic of the magnetic moments (arrows) distribution in nanotwined films for different relations between the exchange correlation length *δ* and the twin variant width *l*: $\delta \ll l$ (**a**), *δ* > *l* (**b**) and *l* ~ *δ* (**c**). Double side arrows denote the magnetocrystalline anisotropy easy axis directions in twin variants. The Inset presents a typical FMR spectrum observed in the film at T = 290 K under magnetic field perpendicular-to-the-film-plane (solid line). Calculated spectra for the cases of strong exchange coupling between twin variants (dash-dot line) and no coupling (dot line) are shown, for comparison.

**Figure 5:**
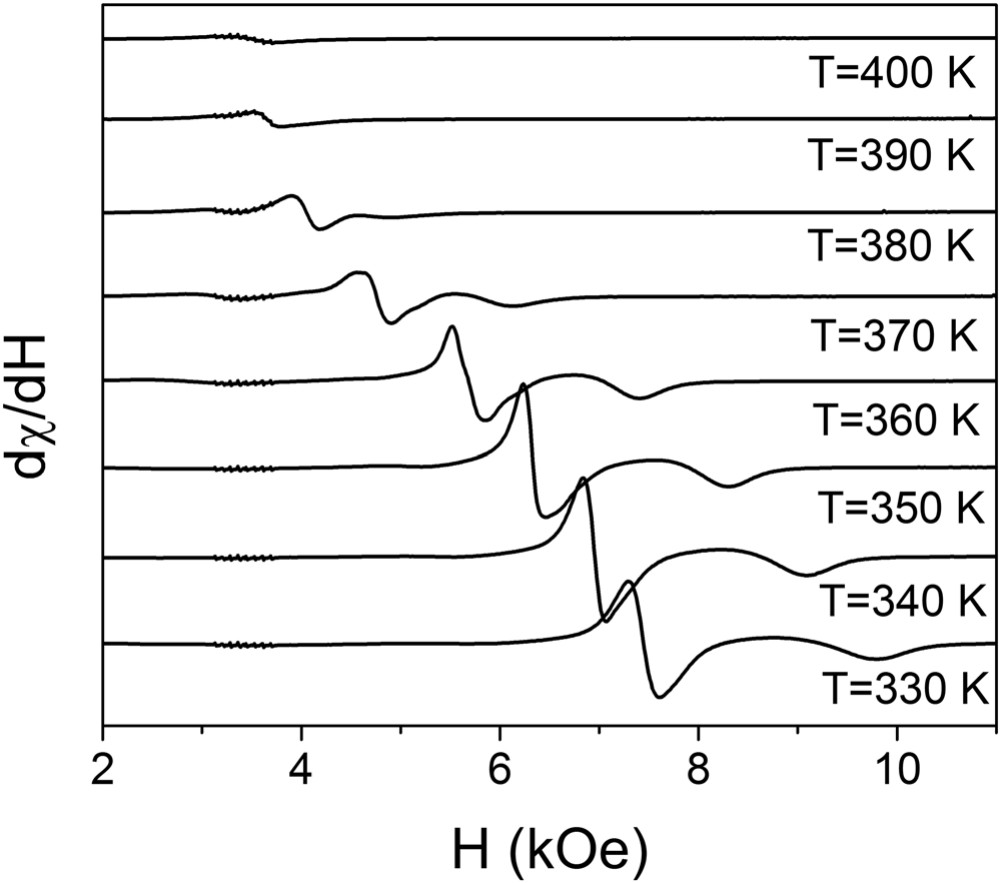
The FMR spectra measured at various temperatures.

Taking into account an irrelevance of the mentioned factors, the appearance of spin disorder in the studied film can be attributed to the formation of stripe-like nanotwin (mesoscale) structure. Previously we discussed this issue in ref.^19^ for similar epitaxial NiMnGa film with a thickness of 500 nm. It has been shown there that the twin width *l* is comparable with the exchange correlation length (domain wall width), $\delta  \sim a{({H}_{E}/{H}_{A})}^{1/2}/2$, where *a* is the lattice parameter, *H*_*E*_ and *H*_*A*_ are the internal magnetic fields that characterize the spin exchange interaction and the magnetocrystalline anisotropy, respectively. The rough estimation of the exchange correlation length (7–19 nm) was performed for single variant martensite using the existed experimental data and theoretical calculations. Although the mentioned theory can’t be directly applied to calculate *δ* for exchange coupled twins it is safe to state that this value should be higher due to the effective anisotropy field reduction^19^, up to 30 nm. Therefore the exchange correlation length in our 300 nm film is also comparable with the twin width (*l* ≈ 60 nm). This statement is confirmed by the shape of ferromagnetic resonance spectra in perpendicular geometry (the magnetic field perpendicular to the film plane). The typical resonance spectrum is presented in the Inset to Fig. 4. This spectrum does not qualitatively modify with the temperature, see Fig. 5.

When the exchange correlation length is smaller than a twin width ($\delta \ll l$), the magnetization vector in the variants aligns along the in-plane anisotropy easy axes with 90° domain walls confined within the twin boundaries (no coupling between twins), see the schematic in Fig. 4. In this case the magnetic resonance conditions for perpendicular geometry are the same as for single variant films, because the magnetisation in each variant precesses independently. The position of the resonance line was calculated using Eq. (17) from ref.^19^ with the set of magnetic parameters discussed there (green line in the Inset to Fig. 4). The schematic in Fig. 4 also shows that in case *δ* > *l* the magnetization vector in the variants aligns along the in-plane anisotropy easy axes with 90° domain walls confined with the twin boundaries (strong coupling between twins). In this case the in-plane orthorhombic uniaxial anisotropy is partially averaged among the variants, with a corresponding reduction (see Eq. (18) in ref.^19^) of the FMR resonance field (red line in the Inset). Finally, if *δ* is comparable to *l*, a non-collinear magnetic structure should be formed, resulting in inhomogeneous broadening of the resonance line^19^. Indeed, the inhomogeneously broadened line was observed in our FMR experiments (black line in the Inset). It is worth to mention that the twin width for 300 nm thin film (60 nm) was found to be a bit smaller than for 500 nm one (80 nm), leading to the smaller degree of the magnetization inhomogeneity.

The higher the degree of the magnetization inhomogeneity is, the broader line should be observed. A parameter that characterizes the magnetization inhomogeneity is Δ*H*_*p*_^19^, see Fig. 4. Other types of inhomogeneities causing the spread of magnetic parameters, such as the composition, structure and/or residual stress, also lead to the resonance line broadening which can be described by the parameter, Δ*H* (Fig. 4). Figure 4 shows that ${\rm{\Delta }}H\ll {\rm{\Delta }}{H}_{p}$ in the entire investigated temperature range, except for the Curie temperature region.

Δ*H*_*p*_ gradually increases when the temperature decreases, whereas the dependence of Δ*H* on the temperature is nonmonotonous (Fig. 4). An increase of Δ*H* near the Curie temperature is related to the thermal fluctuation of the magnetization when the temperature approaches the magnetic order-disorder region. At low temperatures, the increase of Δ*H* is related to the increase of the magnetization, magnetic anisotropy fields and stresses due to different coefficients of thermal expansion of the film and the substrate, which enhance the inhomogeneities. The increase of Δ*H*_*p*_ during cooling is related to the increase of magnetization variation in the nanotwins, due to the decrease of the exchange correlation length. The latter is produced by the increase of the magnetic anisotropy field^21,22^ at low temperatures^19^.

Apparently, the increase of magnetic inhomogeneity entails an increase of electron scattering in the nanotwin structure. This should result in the increase of magnetoresistance value at low temperatures. However, the higher the magnetization inhomogeneity is, the higher magnetic fields should be applied to align the magnetic moments leading to a decrease of the magnetoresistance in a finite magnetic field. We cannot also discard the decrease of the negative magnetoresistance as a result of the increase of normal (positive) magnetoresistance in the low temperature regime, due to the well-known Lorentz force action on charge carriers. The contribution of this mechanism grows when the temperature decreases, due to the temperature-induced reduction of the electron mean free path. The interplay of these factors results in the appearance of the broad maximum on the temperature dependence of magnetoresistance observed experimentally (Fig. 2).

In conclusion, it is found that the nanotwin structure in the martensitic state of epitaxial ferromagnetic shape memory films results in the formation of a non-collinear magnetic structure, which leads to the negative magnetoresistance observed experimentally in a wide temperature range. This mechanism is the reason of a high value of magnetoresistance in the Heusler alloys with large electron free path. A similar effect is also expected for metamagnetic shape memory alloys with fine twin structures exhibiting ferromagnetic exchange inside the twin variants and antiferromagnetic exchange across twin boundaries, as recently reported in ref.^23^. A remarkable applied aspect of the studied magnetoresistance effect is the possibility of generating spin waves with defined frequencies by electric current pulses on periodic nanotwin structures formed in the MSM epitaxial films. Such an effect can be used in magnonic devices.

## Methods

A 300 nm-thick Ni_52.3_Mn_26.8_Ga_20.9_ (at.%) film was epitaxially grown by magnetron sputtering onto a MgO(001) substrate heated at 500 °C under 2.6 × 10^−2^ mbar pressure and 150 W power. Composition of the film was determined with an uncertainty of 0.5 at.% by energy-dispersive x-ray spectroscopy (EDX), using a scanning electron microscope Jeol JSM-6400. Philips X’Pert PRO x-ray diffractometer (CuK_α_ radiation) was used for the structure investigation in 100–450 K temperature range. The atomic force microscopy (Veeco Nanoscope IVA) was used for the visualization of the twin structure. Magnetic parameters of the films were determined from magnetic (Quantum Design SQUID MPMS-5 magnetometer) and ferromagnetic resonance measurements (Bruker ELEXSYS E500 electron spin resonance spectrometer). The magnetoresistance was measured using a standard four-probe method under magnetic fields up to 9 kOe in the temperature range of 100–370 K.

## References

[1] Felser Claudia, Hirohata Atsufumi (2016). Heusler Alloys.

[2] Heczko O, Sozinov A, Ullakko K (2000). Giant field-induced reversible strain in magnetic shape memory NiMnGa alloy. IEEE Trans. Mag..

[3] Murray SJ, Marioni M, Allen SM, O’Handley RC, Lograsso TA (2000). 6% magnetic-field-induced strain by twin-boundary motion in ferromagnetic Ni–Mn–Ga. Appl. Phys. Lett..

[4] Acet, M., Manosa, L., & Planes, A. Magnetic-field-induced effects in martensitic Heusler-based magnetic shape-memory alloys in *Handbook of Magnetic Materials* (ed. Buschow, K. H. J.) **19**, 231–289 (Elsevier 2011).

[5] Planes A, Manosa L, Acet M (2009). Magnetocaloric effect and its relation to shape-memory properties in ferromagnetic Heusler alloys. J. Phys.: Condens. Mat..

[6] *Disorder and strain-induced complexity in functional materials* (ed. Kakeshita, T., Fukuda, T., Saxena, A. & Planes, A.) **148** (Springer Series in Material Science, Springer-Verlag (2012)).

[7] *Magnetic Nanostructures. Spin Dynamics and Spin Transport* (ed. Zabel, H. & Farle, M.) (Springer-Verlag, (2013).

[8] Baibich MN (1989). Giant Magnetoresistance of (001)Fe/(001)Cr Magnetic Superlattices. Phys. Rev. Lett..

[9] Kakazei GN (1999). Influence of co-evaporation technique on the structural and magnetic properties of CoCu granular films. J. Magn. Magn. Mater..

[10] Kataoka M (2003). Resistivity and magnetoresistance of ferromagnetic metals with localized spins. Phys. Rev. B.

[11] Barandiarán JM, Chernenko VA, Lázpita P, Gutiérrez J, Feuchtwanger J (2009). Effect of martensitic transformation and magnetic field on transport properties of Ni-Mn-Ga and Ni-Fe-Ga Heusler alloys. Phys. Rev. B.

[12] Banik S (2008). Magnetoresistance behavior of ferromagnetic shape memory alloy Ni_1.75_Mn_1.25_Ga. Phys. Rev. B.

[13] Sharma VK, Chattopadhyay MK, Shaeb KHB, Chouhan A, Roy SB (2006). Large magnetoresistance in Ni_50_Mn_34_In_16_ alloy. Appl. Phys. Lett..

[14] Yu SY (2006). Large magnetoresistance in single-crystalline Ni_50_Mn_50−x_In_x_ alloys (x = 14–16) upon martensitic transformation. Appl. Phys. Lett..

[15] Tolea F (2017). Specific Changes in the Magnetoresistance of Ni–Fe–Ga Heusler Alloys Induced by Cu, Co, and Al Substitutions. IEEE Trans. Mag..

[16] Golub VO (2004). Anomalous magnetoresistance in NiMnGa thin films. J. Appl. Phys..

[17] Auge A (2012). Thickness dependence of the martensitic transformation, magnetism, and magnetoresistance in epitaxial Ni-Mn-Sn ultrathin films. Phys. Rev. B.

[18] Srivastava VK, Chatterjee R, O’Handley RC (2006). Effect of twin boundaries on electrical transport in a Ni–Mn–Ga single crystal. Appl. Phys. Lett..

[19] Chernenko VA, Lvov VA, Golub V, Aseguinolaza IR, Barandiarán JM (2011). Magnetic anisotropy of mesoscale-twinned Ni-Mn-Ga thin films. Phys. Rev. B.

[20] Acet M, Wassermann EF (2012). Magnetic interactions in Ni-Mn-based magnetic shape-memory heusler alloys. Adv. Eng. Mater..

[21] Sakon T, Adachi Y, Kanomata T (2013). Magneto-Structural Properties of Ni_2_MnGa Ferromagnetic Shape Memory Alloy in Magnetic Fields. Metals.

[22] L’vov VA (2009). The role of anisotropic thermal expansion of shape memory alloys in their functional properties. Acta Materialia.

[23] Golub VO (2017). Antiferromagnetic coupling between martensitic twin variants observed by magnetic resonance in Ni-Mn-Sn-Co films. Phys. Rev. B.

